# Effects of self-management exercise group participation in community-dwelling older adults

**DOI:** 10.1186/s12877-022-03509-2

**Published:** 2022-10-21

**Authors:** Mieko Yokozuka, Kanako Okazaki, Masayuki Hoshi, Ayumi Shiine, Tomoko Fukumoto

**Affiliations:** 1 Department of Physical Therapy, Fukushima Medical University School of Health Sciences, 10-6 Sakae-machi, Fukushima City, Fukushima 960-8516 Japan; 2 Health Promotion Division, Public Health Center, Health and Welfare Department, 2-15-1 Asahi Koriyama, Koriyama City, Fukushima 963-8024 Japan; 3 Integrated Community Care System Promotion Division, Health and Welfare Department, 1-23-7 Asahi Koriyama, Koriyama City, Fukushima 963-8601 Japan

**Keywords:** Community-dwelling, Exercise group, Older adults, Participation, Self-management

## Abstract

**Background:**

The effects of physical fitness and age on motor function in older adults who continue to exercise remain unclear. This study aimed to examine the effects of participation in self-management exercise groups in adults aged ≥65 years.

**Methods:**

The motor functions of 372 citizens who participated in a self-management exercise group for 1 year were examined. The motor functions were assessed by measuring grip strength, five-repetition sit-to-stand test, 5-m fastest walking time (walking time) and timed up and go test. The participants were grouped according to their baseline grip strength (low or high grip strength groups). The baseline parameters were compared to those assessed 1 year after group participation. In addition, the rates of long-term care/support need certification were examined at 2-year follow-up.

**Results:**

In the low grip strength group aged ≥75 years, the grip strength of men, and grip strength and five-repetition sit-to-stand test results of women improved after 1 year. In the high grip strength group, the five-repetition sit-to-stand and timed up and go test results of men aged 65–74 years and five-repetition sit-to-stand test results of men aged ≥75 years improved. Among women in the high grip strength group, grip strength, five-repetition sit-to-stand test, walking time, and timed up and go test results improved in the participants aged 65–74 and ≥ 75 years. The number of new long-term care/support need certifications was comparable in both groups.

**Conclusions:**

Participation in self-management exercise groups led to maintaining or improving physical fitness among community-dwelling-older adults. Furthermore, higher baseline grip strength was associated with improvements in many motor functions; therefore, participation in self-management exercise groups before the onset of functional decline is desirable.

## Background

In Japan, the long-term care insurance system was established in 2000 to support the long-term care of Japanese citizens, including older adults. Since 2015, self-management groups called “kayoi-no-ba” (a “place for regular visits” in Japanese) have been operated by local residents to prevent the need for long-term care in older adults [1, 2]. From 2013 to 2017, the numbers of self-management groups and participants have increased annually [2]. The characteristics of a self-management group are 1) contributes to the prevention of long-term frailty through exercise, such as hobby activities, 2) operates primarily by municipality residents, 3) municipality-provided financial support may or may not be provided, and 4) activities are offered more than once a month [3]. Therefore, the programs conducted in each self-management group are not standardized. It has been reported that community-based self-management group exercise reduced the incidence of disability during a 4-year follow-up period in community-dwelling older adults [4]; participation in hobby activities enhanced social interactions among the residents, promoting and maintaining cognitive function [5, 6]. It has also been reported that participation in self-management groups increased social participation and desirable changes in psychological health [7]. Self-management groups are operated primarily by local residents, making it difficult to evaluate the effects of these interventions on motor function. Thus, although several studies have reported positive outcomes with the participation in self-management groups on social and cognitive function, few have investigated the effects on motor functions.

Several effects of physical activities on the health of older adults have been reported. These reports included improvements in physical metrics, such as strength, walking speed, and the five times sit-to-stand test results after participation in exercise for 3–6 months. There have also been a few reports of improvements in physical metrics with continued participation in exercise for ≥12 months [8]. Since 2015, Koriyama City has supported the activities of self-management groups through an exercise called “Iki-Iki Hyakusai Taiso” [9] in collaboration with rehabilitation professionals to assist the self-management groups run by the residents. Participation in this type of exercise improved the physical fitness of the participants and increased their participation in social activities after 1 year [10]. The average age of the participants in this report was 72.6 years. Anyone who can travel from their home to the group meeting place can participate in the self-management group run by the community. However, since people with various characteristics can participate, the effect of physical fitness and age on motor functions remains unclear.

The purpose of this study was to compare the effects of continued participation in self-management exercise groups for at least 1 year according to participants’ characteristics, such as physical fitness and age. In addition, as a spillover effect, we compared the number of new certifications for long-term care/support issued due to differences in physical fitness.

## Methods

### Participants

This prospective study included people aged ≥65 years who participated in a self-management exercise group between 2015 and 2018. Motor functions were evaluated at the first attendance and 3 and 12 months after the self-management exercise group was established. A total of 954 people participated in one of the measurements: 132 in 2015, 499 in 2016, and 323 in 2017. Participants underwent at least one motor function test before and 1 year after participation in the exercise group.

### Exercise

Koriyama City’s public health nurses and rehabilitation professionals, employed at medical institutions in the city, support the establishment of self-management exercise groups. Following establishment, the self-management exercise groups are operated independently by city residents. The participants performed the exercise program “Iki-Iki Hyakusai Taiso” [9], created in the exercise studio in Kochi City. The “Iki-Iki Hyakusai Taiso” consists of a warm-up, muscle strength, and cool-down exercises, requiring approximately 30 minutes to complete. The warm-up exercises include deep breathing, shoulder and side stretching, foot stepping, and knee holding exercises. All warm-up exercises were performed sitting on a chair, twice each to the call of 1-8. The muscle strength exercises include raising the arms forward and to the sides, sitting on a chair with weights on the wrists, sitting and standing up from a chair, extending the knees while sitting on a chair with weights on the ankles, and raising the legs sideways, standing with ankle weights. Each exercise involved 10 repetitions, slowly counting from 1 to 8. The weights ranged from 0 to 2.2 kg, assigned to each individual based on their fitness level. The cool-down excises include wrist and arm stretches, thigh stretches, and neck exercises, without the weights; the participants sat on a chair for 15 seconds each exercise. It is recommended that the participants perform these exercises at least once a week.

### Evaluation of motor functions

Dementia, cerebrovascular accidents, and age-related frailty are the main causes of long-term care need [11]. Declining grip strength is associated with the risk of developing dementia [12] and cerebrovascular accidents [13]. In addition, the measurement of grip strength is one of the five items of the revised Japanese version of the Cardiovascular Health Study criteria (J-CHS criteria), which is used to assess frailty syndrome [14]. Therefore, we compared the effects of participation in the exercise program on grip strength as an index of physical fitness.

Motor functions were measured at the start of “Iki-Iki Hyakusai Taiso” and 3 and 12 months thereafter, on days determined by each community group. Participant information, including name, address, and date of birth, was obtained to observe their progress. Measurements were taken by physical therapists, occupational therapists, public health nurses, community nurses, and other professionals. A manual was prepared, and measurements were taken according to the description to ensure a uniform assessment method. Grip strength and the five-repetition sit-to-stand (5R-STS) test were used to evaluate muscle strength. The 5-m fastest walking time (walking time) and timed up-and-go (TUG) test were used to evaluate mobility.

Grip strength was measured once on each side, with both upper limbs hanging down in a standing position. During the 5R-STS test, participants were instructed to repeat the motions of standing from and sitting on a chair with their arms folded in front of their chest and feet spread shoulder-width apart. The total time required for five stand/sit cycle repetitions was recorded. Walking time was measured by instructing the participants to walk a path of five meters, walk one meter in front and behind for seven meters as fast as possible and record the time required to traverse the 5-m path. The TUG test measured the time required for the participant to stand up from a chair, walk to a landmark three meters away, return to the chair, and sit. All the measurements were taken once.

### Certification of need for long-term care/support

We investigated whether the participants received certification of the need for long-term care/support before participating in the exercise group and whether there was a new certification 2 years from April of the year when they participated in the exercise group to March of the following year.

### Statistical analysis

According to the revised J-CHS criteria, a grip strength of < 28 kg for men and that of < 18 kg for women was considered weak. The participants were classified according to the grip strength of both hands as follows: those with grip strength in both hands that was less than the standard value (e.g., < 28 kg for men and < 18 kg for women) were placed in the low grip strength group, and those with a grip strength greater than or equal to the standard value (e.g., ≥ 28 kg for men and ≥ 18 kg for women) in one or both hands were placed in the high grip strength group.

In each group, motor functions before participation and 1 year after participation in the exercise group were compared by sex and age group. The normality of motor function variable distribution at baseline and 1 year after participation was examined by the Shapiro-Wilk test. Comparisons were made with the paired t-test and Wilcoxon signed rank-sum test for normally and non-normally distributed variables, respectively. The chi-squared test was used to compare the frequencies of long-term care/support need certifications according to the new applications following participation in the exercise group, assessed at the start of participation and every year for the 2 subsequent years. Statistical analyses were performed using SPSS version 28 (IBM Corp., Armonk, NY, USA) with a significance level of < 5%.

### Ethical considerations

This study was a joint research project to promote the Sustainable Development Goals Experience Future City Koriyama All-Generation Healthy Urban Area Creation Project and was approved by the Fukushima Medical University Ethics Committee (2021-096). This study complied with data protection protocols established with Koriyama City and used anonymized data received from Koriyama City. Therefore, there was no need to obtain informed consent or disclose information.

## Results

This analysis included 63 people who participated in 2015 (15 men and 48 women), 194 in 2016 (39 men and 155 women), and 115 in 2017 (27 men, 88 women), with a total of 372 participants.

### Changes in motor functions after one year according to grip strength

Table 1 shows the changes in motor functions after 1 year due to differences in grip strength in participants aged ≥65 years. The low grip strength group does not include men or women aged 65–74 years. In the participants aged ≥75 years, grip strength (R) (*P* = 0.009) of men and grip strength (L) (*P* = 0.021) and 5R-STS test results (*P* < 0.001) of women showed significant improvement at 1 year, compared to baseline values.

**Table 1:** Changes in motor function after one year by grip strength

		Low-value group					High-value group				
		pre		post 1y		*p*-value	pre		post 1y		*p*-value
Men	65-74 years old	*n* = 0					*n* = 32				
	Grip strength (R)	–		–		–	32.3	± 3.9	32.9	± 4.1	0.300
	Grip strength (L)	–		–		–	31.5	± 3.4	31.6	± 3.5	0.825
	5R-STS	–		–		–	8.4	(7.1 - 9.5)	7.7	(6.3 - 8.5)	0.002
	Walking time	–		–		–	2.7	(2.6 - 3.0)	2.6	(2.5 - 2.9)	0.052
	TUG	–		–		–	6.0	± 1.1	5.5	± 1.4	0.013
	75 years and over	*n* = 22					*n* = 27				
	Grip strength (R)	24.1	± 2.3	26.3	± 3.4	0.009	30.5	(28.3 - 32.0)	30.5	(28.8 - 32.9)	0.445
	Grip strength (L)	24.1	± 2.5	25.0	± 3.9	0.198	29.3	(26.1 - 30.4)	29.5	(27.1 - 30.4)	0.169
	5R-STS	11.4	(10.1 - 14.2)	9.4	(8.8 - 11.3)	0.122	9.9	± 2.0	8.7	± 1.9	0.001
	Walking time	2.8	(2.5 - 3.5)	2.8	(2.6 - 3.2)	0.572	2.8	(2.6 - 3.0)	2.7	(2.3 - 3.0)	0.068
	TUG	6.9	(5.7 - 8.4)	6.7	(6.2 - 7.0)	0.820	6.7	± 1.5	6.7	± 1.7	0.925
Women	65-74 years old	*n* = 0					*n* = 115				
	Grip strength (R)	–		–		–	22.5	(20.5 - 24.8)	23.0	(21.5 - 25.0)	0.001
	Grip strength (L)	–		–		–	21.7	± 2.7	22.2	± 2.6	0.007
	5R-STS	–		–		–	8.7	(7.9 - 10.2)	7.4	(6.2 - 8.2)	< 0.001
	Walking time	–		–		–	2.7	(2.5 - 3.0)	2.6	(2.4 - 2.9)	0.001
	TUG	–		–		–	5.9	(5.2 - 6.4)	5.5	(5.1 - 6.1)	< 0.001
	75 years and over	*n* = 57					*n* = 119				
	Grip strength (R)	15.5	(14.0 - 16.5)	16.0	(13.5 - 18.0)	0.240	20.5	(19.0 - 22.0)	20.5	(19.0 - 23.0)	0.652
	Grip strength (L)	14.0	(12.1 - 16.0)	14.5	(12.5 - 17.5)	0.021	19.1	± 2.5	19.5	± 2.6	0.008
	5R-STS	10.2	(9.1 - 13.9)	9.2	(7.2 - 10.9)	< 0.001	9.6	(8.5 - 11.5)	8.7	(6.7 - 9.9)	< 0.001
	Walking time	3.4	(2.9 - 4.3)	3.3	(2.9 - 4.1)	0.476	3.2	(2.8 - 4.0)	3.0	(2.8 - 3.5)	< 0.001
	TUG	7.7	(6.8 - 9.5)	7.1	(6.2 - 8.6)	0.108	6.8	(6.0 - 7.6)	6.3	(5.8 - 7.3)	0.015

Unit: Grip strength (kg), 5R-STS, Walking time, and TUG (sec)

Median (interquartile range) or mean ± standard deviation

In the high grip strength group, the 5R-STS test (*P* = 0.002, < 0.001) and TUG test (*P* = 0.013, < 0.001) results in both men and women aged 65–74 years improved, relative to the baseline values. In addition, grip strength (R: *P* = 0.001, L: *P* = 0.007) and walking time (*P* = 0.001) of women showed significant improvement. In participants aged ≥75 years, the 5R-STS test (men: *P* = 0.001, women: *P* < 0.001) improved significantly, relative to the baseline values. In addition, grip strength (L) (*P* = 0.008), walking time (*P* < 0.001), and TUG test (*P* = 0.015) of women aged ≥75 years improved significantly, relative to the baseline values.

### Comparison of the certification of the need for long-term care/support

Table 2 shows the long-term care/support need certification rate according to grip strength in participants aged ≥75 years. Among participants aged ≥75 years at the time of initiating participation in the exercise group, 11 people in the low grip strength group and eight in the high grip strength group were certified as needing long-term care/support, while 68 people in the low grip strength group and 138 in high grip strength group were not certified. There was a significant difference between those rates (*p* = 0.030).

**Table 2:** Long-term care/support need certification rates by grip strength in participants aged ≥75 years

	Low-value group	High-value group	*p*-value
Pre	11/68	8/138	0.030
1 year Post	2/66	7/131	0.482
2 years Post	4/62	4/127	0.313

Unit: Values are presented as counts (need/no need)

Among participants who did not have a long-term care/support need certification at baseline, two in the low-strength group and seven in the high-strength group received new certifications within 1 year from joining the program; in addition, another four participants in each group received this certification within 2 years. The new certification rates were comparable between the groups.

## Discussion

There were no participants aged 65–74 years who had grip strength below the standard value when initiating participation in the exercise group. In individuals with grip strengths below the standard value (aged ≥75 years), continued exercise for 1 year improved their grip strength. Continued exercise for 1 year also improved the 5R-STS test results in women in the low grip strength group. Furthermore, after 1 year of continued exercise participation, the 5R-STS test improved in participants aged ≥75 years in the high grip strength group. Women aged ≥75 years in the high grip strength group demonstrated improvements in grip strength, walking time, and the TUG test following 1 year of continued exercise participation.

Several studies have reported the effects of exercise in people aged ≥75 years. In people with an average age of 83.3 years, frailty and mobility disability can be successfully treated using an interdisciplinary, multifaceted treatment program [15]. In addition, it has been reported that resistance exercise, twice a week for 8 weeks, influenced muscle strength and motor functions in people aged 80 to 88 years [16]. Similarly, the present study found that motor functions improved with the continued participation in the exercise program, even in persons aged ≥75 years. In particular, the 5R-STS test results improved in participants with baseline grip strengths that exceeded the standard value. Concerning the relationship between maximal isometric strength and mobility at 75 years of age, it has been reported that greater grip strength was associated with higher mobility in women [17]. In this study, greater grip strength and associated higher mobility may have been maintained during the study period. Therefore, it is presumed that, even when participating in the exercise program, the weight used in the standing exercise was high, and engagement in activities of daily living, other than participation in the exercise group, was high among the study participants.

There was no significant difference in the rates of new long-term care/support need certification according to baseline grip strength in participants aged ≥75 years; several studies have reported that 4 years of continued participation in exercise reduces this rate in frail older adults [4]. The average age of frail older adults included in the above study was approximately 80 years [4]. Other studies have found that participation in volunteer activities by community-dwelling older adults for the prevention of disability reduced the rate of “mild” long-term care/support need certifications in the region [18]. An increase in the number of long-term care/support need certifications has been reported for individuals aged ≥75 years [19]; the causative diseases include malignant tumors, joint disease, and dementia [20]. People aged ≥75 years may require help with household chores due to functional deterioration and long-term care due to the onset of illnesses. Thus, this age group may apply for applicable certification more often than the younger age groups. This study also suggested that certain factors, including the occurrence of illness, may be more influential on the long-term care requirements than differences in physical fitness.

Through participation in group activities, the self-management exercise groups presented an opportunity for social interaction for older adults of all age groups. Although various activities are conducted in self-management exercise groups, this study demonstrated that participating in the exercise group led to maintaining or improving physical fitness. Furthermore, higher baseline grip strength values were associated with improvements in many motor functions; therefore, participation in self-management exercise groups before the onset of functional decline is desirable.

This study has some limitations. First, the self-management exercise groups were a resident-led initiative conducted at least once weekly. Koriyama City supports the start-up of self-management exercise group activities and physical fitness measurements. Therefore, the state of activities prior to participation in self-management exercise groups, the frequencies of the exercises, and each individual’s participation and other activities are unknown. We could not determine differences in effect according to the frequency of exercise. Second, the standard values of grip strength used to assess frailty, as defined by the revised J-CHS criteria, do not differ by age. Therefore, because grip strength declines with age, adults aged ≥75 years were more likely to be categorized in the low grip strength group. Third, the cause of the new certification of the need for long-term care/support was unclear. It is necessary to examine the physical and cognitive functions and illnesses of those certified to need long-term care/support while participating in the self-management exercise groups. Fourth, we could not evaluate the number of participants who ceased participation in self-management exercise group activities or the reason for ceasing participation. It is unclear whether individuals ceased participation in self-management exercise group activities due to the onset of illness or injury and whether these individuals were certified as needing long-term care/support. In future studies, it will be prudent to monitor the participants who discontinue program participation to identify the reasons for discontinuation.

## Conclusions

The effects of participation in self-management exercise group activities for 1 year on the motor function of older adults were examined according to baseline physical fitness levels, age, and sex. The participants were categorized into low and high grip strength groups by baseline grip strength according to grip strength standards defined by the revised J-CHS criteria. In the low grip strength group with participants aged ≥75 years, grip strength improved in men, and grip strength and 5R-STS test results improved in women, following a year of participation in self-management exercise group activities. In the high grip strength group, among men aged 65–74 years, the 5R-STS and TUG test results improved, while among men aged ≥75 years, only the 5R-STS test results improved. Furthermore, in the high grip strength group, among women aged 65–74 and ≥ 75 years, grip strength, 5R-STS test, walking time, and TUG test results improved. The certification rate for the need for long-term care/support within 2 years of participation in the self-management exercise group program did not differ according to baseline grip strength.

## Data Availability

This study was a joint research project with Koriyama City. Data provided by Koriyama City cannot be shared publicly because of a lack of approval to publish.

## Abbreviations

J-CHS: Japanese version of the Cardiovascular Health Study
5R-STS: Five-repetition sit-to-stand
TUG: Timed up and go

## References

[1] Ministry of Health, Labor and Welfare. Guidance for promoting long-term care prevention through community development [original article in Japanese]. Tokyo, Japan: Ministry of Health, Labor and Welfare. https://www.mhlw.go.jp/file/06-Seisakujouhou-12300000-Roukenkyoku/0000122064.pdf Accessed 30 Nov 2021.

[2] Yamada M, Arai H (2020). Long-term care system in Japan. Ann Geriatr Med Res.

[3] Ministry of Health, Labor and Welfare. Community long-term care prevention activities [original article in Japanese]. Tokyo, Japan: Ministry of Health, Labor and Welfare. https://www.mhlw.go.jp/content/12601000/000529367.pdf Accessed 30 Nov 2021.

[4] Yamada M, Arai H (2017). Self-management group exercise extends healthy life expectancy in frail community-dwelling older adults. Int J Environ Res Public Health.

[5] Hikichi H, Kondo N, Kondo K, Aida J, Takeda T, Kawachi I (2015). Effect of a community intervention programme promoting social interactions on functional disability prevention for older adults: propensity score matching and instrumental variable analyses, JAGES Taketoyo study. J Epidemiol Community Health.

[6] Hikichi H, Kondo K, Takeda T, Kawachi I (2017). Social interaction and cognitive decline: results of a 7-year community intervention. Alzheimers Dement (N Y).

[7] Hayashi T, Takeda T, Kato K, Kondo K (2019). Association between subjective changes in social participation and those in the health information they receive and health awareness among participants in “Kayoino-Ba”: JAGES survey of participants in “Kayoino-Ba”. Gen Rehabil.

[8] Haider S, Grabovac I, Dorner TE (2019). Effects of physical activity interventions in frail and prefrail community-dwelling people on frailty status, muscle strength, physical performance and muscle mass-a narrative review. Wien Klin Wochenschr.

[9] Pamphlet about Iki-Iki Hyakusai Taiso [original article in Japanese]. Kochi City, Kochi, Japan. https://www.city.kochi.kochi.jp/uploaded/attachment/116290.pdf Accessed 15 May 2022.

[10] Tuo J, Kabayama M, Huang Y, Akagi Y, Godai K, Kiyoshige E (2021). Effect of Iki-Iki Hyakusai Taiso on the prevention of progression of frailty in Nose Town, Osaka Prefecture. Nihon Ronen Igakkai Zasshi.

[11] Ministry of Health, Labor and Welfare. Comprehensive survey of living conditions: percentage distribution of major causes (Top 3 causes) of long-term care by the present care requirement level [original article in Japanese]. Tokyo, Japan: Ministry of Health, Labor and Welfare. https://www.mhlw.go.jp/english/database/db-hss/cslc-index.html Accessed 30 Nov 2021.

[12] Bullain SS, Corrada MM, Shah BA, Mozaffar FH, Panzenboeck M, Kawas CH (2013). Poor physical performance and dementia in the oldest old: the 90+ study. JAMA Neurol.

[13] Leong DP, Teo KK, Rangarajan S, Lopez-Jaramillo P, Avezum A, Orlandini A (2015). Prognostic value of grip strength: findings from the Prospective Urban Rural Epidemiology (PURE) study. Lancet..

[14] Satake S, Arai H (2020). The revised Japanese version of the cardiovascular Health Study criteria (revised J-CHS criteria). Geriatr Gerontol Int.

[15] Cameron ID, Fairhall N, Langron C, Lockwood K, Monaghan N, Aggar C (2013). A multifactorial interdisciplinary intervention reduces frailty in older people: randomized trial. BMC Med.

[16] Kalapotharakos VI, Diamantopoulos K, Tokmakidis SP (2010). Effects of resistance training and detraining on muscle strength and functional performance of older adults aged 80 to 88 years. Aging Clin Exp Res.

[17] Rantanen T, Era P, Heikkinen E (1994). Maximal isometric strength and mobility among 75-year-old men and women. Age Ageing.

[18] Kozawa T, Tanaka K, Seino S, Yamada D, Omori Y, Ota H (2014). Volunteer activities by community-dwelling older adults for the prevention of disability may reduce the rate of long-term care eligibility. Jpn J Health Promot.

[19] Cabinet Office. Annual Report on the Ageing Society: 2018 (Summary), Chapter 1 Situation on Ageing Population, Section 2 Trends of Living of the Elderly. Tokyo, Japan: Cabinet Office. https://www8.cao.go.jp/kourei/english/annualreport/2018/pdf/c1-2-1.pdf Accessed 25 May 2022.

[20] Takahashi K, Tsukishima E (2017). Diseases requiring severe-level care certification for long-term care insurance. Nihon Koshu Eisei Zasshi.

